# Breathable and Stretchable Organic Electrochemical Transistors with Laminated Porous Structures for Glucose Sensing

**DOI:** 10.3390/s23156910

**Published:** 2023-08-03

**Authors:** Haihong Guo, Changjian Liu, Yujie Peng, Lin Gao, Junsheng Yu

**Affiliations:** State Key Laboratory of Electronic Thin Films and Integrated Devices, School of Optoelectronic Science and Engineering, University of Electronic Science and Technology of China (UESTC), Chengdu 610054, China; 202221050501@std.uestc.edu.cn (H.G.); 202121050109@std.uestc.edu.cn (C.L.); 202211050804@std.uestc.edu.cn (Y.P.); 202111050704@std.uestc.edu.cn (L.G.)

**Keywords:** porous structure, stretchable and breathable electronics, organic electrochemical transistors, glucose sensors

## Abstract

Dynamic glucose monitoring is important to reduce the risk of metabolic diseases such as diabetes. Wearable biosensors based on organic electrochemical transistors (OECTs) have been developed due to their excellent signal amplification capabilities and biocompatibility. However, traditional wearable biosensors are fabricated on flat substrates with limited gas permeability, resulting in the inefficient evaporation of sweat, reduced wear comfort, and increased risk of inflammation. Here, we proposed breathable OECT-based glucose sensors by designing a porous structure to realize optimal breathable and stretchable properties. The gas permeability of the device and the relationship between electrical properties under different tensile strains were carefully investigated. The OECTs exhibit exceptional electrical properties (g_m_ ~1.51 mS and I_on_ ~0.37 mA) and can retain up to about 44% of their initial performance even at 30% stretching. Furthermore, obvious responses to glucose have been demonstrated in a wide range of concentrations (10^−7^–10^−4^ M) even under 30% strain, where the normalized response to 10^−4^ M is 26% and 21% for the pristine sensor and under 30% strain, respectively. This work offers a new strategy for developing advanced breathable and wearable bioelectronics.

## 1. Introduction

Glucose, the primary source of energy in biological systems, needs to be regularly assayed for its concentration in blood as a vital physiological indicator of the human body [1,2]. While the detection of glucose in sweat or cellular fluid can be accomplished through liquid chromatography and optical methods, these approaches necessitate the utilization of complex and expensive equipment [3,4]. Consequently, the development of rapid and cost-effective chemical sensors for glucose detection is highly necessary. In tandem with recent developments in on-skin and implantable electronics, biosensors that are tightly integrated with the human body have been intensively explored in real-time monitoring and biological and diagnostic applications, especially in physiological health testing [5,6,7]. The utilization of transistor-type active components as active electrodes for the local amplification of bio-potential signals has been implemented to enhance the signal quality. This approach effectively reduces the output impedance and suppresses noise, thereby improving the overall signal quality [8]. This approach has been implemented in ion sensors [9], biomolecules sensors (e.g., virus sensing, neurotransmitter sensing, enzymatic sensing), and electrophysiological and electromyographic monitoring [10,11,12].

The most effective approach for measuring chemical or bioelectrical signals is achieved by directly placing flexible OECTs in contact with the skin surface. This strategy enhances the human–machine interface contact, facilitates high-fidelity signal transmission, and enables real-time monitoring [13,14]. Extensive research efforts have been dedicated to endow high-performance OECTs with flexible and wearable properties. For example, Chen et al. reported a prestretched transferring electrode and an active layer for stretchable OECTs exhibiting stable signal amplification for electrocardiogram and a simulated synapse response under 60% mechanical deformation [15]. Demuru et al. utilized an antibody-coated gold gate and combined it with microfluidics to realize a limit of detection of 100 pM cortisol [16]. Nevertheless, the numerous existing achievements in flexible or stretchable OECTs ignore comfort and breathability, and are usually fabricated directly on the impermeable elastic substrate. Long-term wear of impermeable devices may result in an elevated local temperature, increased moisture accumulation on the body surface, and the growth of bacteria, which in turn leads to discomfort, inflammation, and potential health risks [17]. This is caused by the lack of a sufficient gas permeable structure; thus, skin is covered with thermal moisture and sweat. On the other hand, skin may be susceptible to scratching when exposed to unsuitable substrates in certain scenarios [18]. As a result, it is imperative to develop a wearable glucose sensor that possesses breathability and stretchability.

In this article, we report an innovative approach to developing breathable and flexible OECTs for glucose-sensing applications. By integrating multiscale porous poly(ethylene-ran-butylene)-block-polystyrene (SEBS) substrates and a porous poly(3-hexylthiophene-2,5-diyl)/poly(ethylene-ran-butylene)-block-polystyrene (P3HT/SEBS) active layer, the OECTs achieve remarkable breathability and stretchability, significantly improving user comfort during daily wear. In addition, the glucose sensor reacts specifically to glucose through the utilization of redox enzyme glucose oxidase (GOx), which is stored in a reservoir of room temperature ionic liquid (RTIL). We thoroughly investigated the effect of different stretching states and varying concentrations of glucose on the electrical performance and show that the transistor enables glucose-sensing applications. Notably, these outcomes have profound implications for the domain of daily health monitoring, underscoring their pivotal role in advancing the development of next-generation wearable electronics.

## 2. Experimental Section

### 2.1. Materials

P3HT with a molecular weight of 10,000 to 100,000 was obtained from Xi’an Polymer Light Technology Corp. SEBS with a molecular weight of approximately 118,000 and poly(vinyl alcohol) (PVA) with a molecular weight of approximately 13,000 to 23,000 (87–89% hydrolyzed) were acquired from Sigma-Aldrich. D-(+)-Glucose, uric acid, ascorbic acid, and GOx (>50 U/mg) were purchased from Aladdin, and 1-Ethyl-3-methylimidazolium bis(trifluoromethylsulfonyl)imide (EMIM:TFSI) and [bis(n5-cyclopentandienyl) iron] (Fc) were obtained from Henwns. Artificial sweat was purchased from Dongguan Chuangfeng Automation Technology Corp. Solvents (chloroform, methanol, isopropyl alcohol (IPA), and toluene) were procured from Tokyo Chemical Industry Company. PBS tablets were sourced from Beijing Solarbio Science & Technology Ltd. (Beijing, China).

### 2.2. Fabrication of Porous SEBS Substrate

The SEBS powders were initially dissolved in chloroform at a concentration of 60 mg/mL (Figure S1a). Subsequently, IPA was introduced into the solution in a volume ratio of 5:2 (chloroform/IPA). The resulting mixture was then subjected to sonication for a duration of 30 min. To form the multiscale porous substrate, 5 mL of the obtained precursor solution was deposited on a 10 × 10 cm^2^ flat aluminum foil, resulting in a membrane with an 8 cm diameter. The membrane was left to dry overnight under ambient conditions, leading to the formation of a multi-scale porous SEBS film with a thickness of 200 μm (Figure S2). Afterward, the porous SEBS film was carefully peeled off the aluminum foil and cut into 2 × 2 cm^2^ squares, which were utilized as substrates for the OECT device.

### 2.3. Fabrication of Active Film

Ultrathin glass (0.15 mm) was first sonicated in IPA for 15 min, followed by drying at 80 °C in an oven. A sacrificial layer of PVA (20 mg/mL in deionized water) was then spin-coated onto UV–ozone treated glass at 3000 rpm for 60 s, followed by annealing at 110 °C for 2 min. P3HT and SEBS were dissolved in chloroform with concentrations of 5 and 2.5 mg/mL, respectively (Figure S1b). Subsequently, methanol was added to the P3HT/SEBS solution at a volume ratio of 92:8 (chloroform/methanol). The resulting mixture was then stirred for 2 h to ensure it was fully dissolved. The addition of methanol serves to form a better porous structure. Next, the mixture was spin-coated onto the sacrificial layer at 88% relative humidity (RH) at 5000 rpm for 10 s. At high RH, the low boiling organic solvent evaporates and cools the glass substrate and vapor during the spin coating process, leading to condensation droplets on the substrate and the formation of porous arrays [19,20]. Finally, the glass with the porous film was immersed in deionized water to dissolve the sacrificial layer, releasing the porous active film on the water’s surface.

### 2.4. Fabrication of the Stretchable and Breathable OECTs

The source and drain electrodes were fabricated by thermal evaporation (60 nm gold) onto a porous SEBS substrate with a shadow mask, where the channel length was set to 50 μm and width to 1.5 mm. Subsequently, the active layer was carefully transferred onto the porous substrate and the residual water was blown off with nitrogen gas.

### 2.5. Characterizations

#### 2.5.1. Electrical Properties

The surface morphology of the substrate, active layer, and OECTs was characterized by field emission scanning electron microscopy (ZEISS Gemini 300, ZEISS, Oberkochen, German). An Agilent B1500 (Keysight, Santa Rosa, CA, USA) semiconductor analyzer was used to test the fundamental electrical properties and glucose sensing performance of the OECTs. During the glucose sensing characterization, the input pulse was generated by a signal generator (DG1022Z, RIGOL Technologies Inc., Beijing, China).

#### 2.5.2. Gas Permeability

The mass of water was carefully controlled at 4000 mg and the transmission rate of water vapor was measured at 30 °C by covering an 0.8 cm diameter beaker with a blank, the gas permeable substrate, the dense substrate, and the OECT devices, respectively. Note that the dense substrate was prepared by dissolving SEBS in toluene (60 mg/mL) and evaporating overnight under atmospheric conditions.

#### 2.5.3. Glucose Detection

A mixture of EMIM:TFSI, GOx (500 units per mL) and Fc (10 mM) was used as the electrolyte (Figure S1c). First, 0.5 μL of the electrolyte was added onto the channel region and an Ag/AgCl reference was used as a gate electrode (V_G_ = −0.7 V). After the drain source current (I_DS_) was constant, 1 μL of PBS solution (1×) containing different concentrations of glucose was added and the variation in I_DS_ was monitored over a period of 60 s. Note, the mixture of PBS and EMIM:TFSI did not spill due to the surface tension in the liquid.

## 3. Results and Discussion

### 3.1. Fabrication and Characterization of the Breathable OECTs

**Figure 1:**
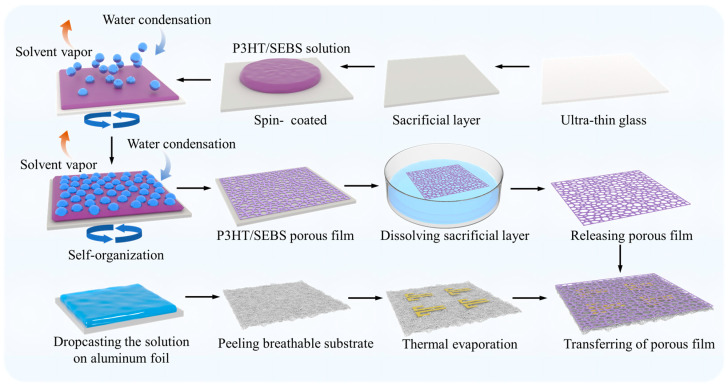
Illustration of the manufacturing process of breathable OECTs.

The detailed manufacturing process is presented in Figure 1. The SEBS elastomer was used as substrate material due to its solution processability and high tensile properties. A breathable substrate was prepared by dropping a precursor solution of the SEBS elastomer and IPA in chloroform onto the aluminum foil under atmospheric conditions. The rapid evaporation of low boiling point chloroform (61.3 °C) in the precursor solution leads to phase separation of high boiling point IPA (82.6 °C) from SEBS to produce nano/micron droplets. As the IPA droplets evaporate, they ultimately form a gas-permeable SEBS substrate [21]. To enhance the stretchability of the device, previous studies have shown that the introduction of SEBS and a porous structure can significantly improve stretchability [22,23,24]. Initially, porous films were fabricated by spin-coating the mixture of P3HT and SEBS in chloroform/methanol onto an ultra-thin glass substrate (0.15 mm) containing a PVA sacrificial layer in an environment with 88% RH. Porous active layers are formed by the breath figure method, where endothermic evaporation of the methanol solvent leads to cooling of the solution surface, triggering a mechanism of water condensation and nucleation in humid air. The temperature gradient between the surface and the SEBS/P3HT solution leads to thermocapillary and Marangani conventions, which drives the self-assembly of the water droplets [25]. In addition, insolubility between water and the P3HT/SEBS solution also contributed to the formation of water droplets on the solution surface. After complete evaporation of the P3HT/SEBS solution and water droplets, the porous structure was formed on the sacrificial layer. Next, the substrate was immersed in deionized water to dissolve the sacrificial layer and release the P3HT/SEBS porous film onto the water’s surface. Finally, the P3HT/SEBS porous film was transferred to the breathable SEBS substrate with prefabricated Au source/drain electrodes (thickness of 60 nm). It is worth noting that the increase in the weight ratio of SEBS contributes to a decrease in the electrical properties of the OECTs, as shown by gradual reductions in on-state currents (I_on_) and transconductance (g_m_) [26]. According to our previous reports, the mixing ratio of P3HT/SEBS was fixed at 2:1 to realize the balance of electrical and mechanical performance [27]. This specific ratio was determined to be optimal for achieving the desired characteristics in this work.

**Figure 2:**
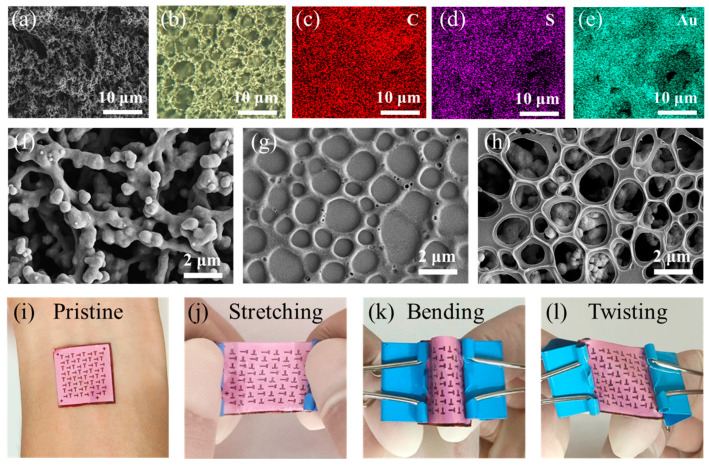
Breathable and stretchable OECTs via a stacking strategy. (**a**) Cross-sectional SEM image of the SEBS substrate; (**b**) optical images of the Au electrode on the SEBS substrate; (**c**–**e**) elemental mapping images of OECTs (S elements from P3HT, C elements from P3HT and SEBS, and Au elements from Au electrode); (**f**–**h**) SEM images of the 3D network Au electrode/substrate, porous P3HT/SEBS films, and breathable OECTs, respectively; (**i**–**l**) optical images of breathable OECT under different mechanical deformations, including skin contact (**i**), stretching (**j**), bending (**k**), and twisting (**l**).

The nano/micron porous stretchable OECTs were realized by laminating a SEBS/P3HT active layer on a multiscale porous SEBS substrate. As shown in Figure 2a, due to phase separation during solvent evaporation, fully interconnected pores appear on the SEBS substrate. Figure 2b also shows that the Au electrode adheres well to the SEBS substrate, forming a 3D network structure and having no effect on the original 3D network structure, thus ensuring the excellent gas permeability of the device. In addition, the elemental distributions in Figure 2c–e show that the P3HT/SEBS active layer can be successfully laminated vertically on the 3D network electrode/substrate. Figure 2f–h illustrates the morphology of the 3D network Au electrode/substrate, P3HT/SEBS porous film, and OECTs devices by SEM, respectively. The SEM images of the 3D network electrode/substrate indicate fully interconnected multiscale pore structures ranging in feature size from approximately 0.2 to 3 μm. Significant holes were observed in the surface morphology of the P3HT/SEBS film with pore sizes of 0.2 to 2 μm, which greatly increases the surface-to-volume ratio and facilitates ion injection [26]. After transfer of the printing process, these devices exhibit porous structures where the P3HT/SEBS porous film has no significant collapses or fractures, maintaining an excellent gas permeability without sacrificing their electrical properties. In addition, Figure 2i–l demonstrates the mechanical robustness of the fabricated devices under various deformations. The incorporation of a multiscale porous substrate and a rubber-elastomer-doped active film confers exceptional flexibility and stretchability to the OECTs. This unique combination ensures the devices remain undamaged under complex deformations such as bending and twisting, even tensile strain. Moreover, the low Young’s modulus of the multiscale porous SEBS substrate, along with the breathable properties of device, allows for conformal contact with the skin. These results highlight the promising potential of these devices for wearable electronics applications.

### 3.2. Electrical and Mechanical Performance of Breathable OECTs

**Figure 3:**
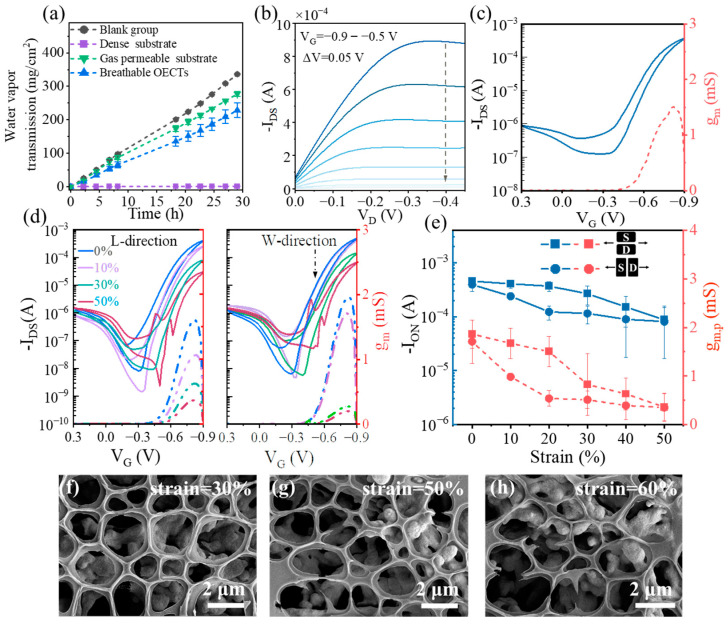
Electrical and mechanical properties of breathable OECTs. (**a**) Water vapor transmission versus elapsed time for blank group (brown), dense substrate (purple), gas permeable substrate (green), and breathable OECTs (blue); output curves (**b**) and transfer curves (**c**) of OECTs; (**d**) transfer curves (solid lines) and corresponding g_m_ evolutions (dashed lines) for the breathable OECTs under variable tensional strain (strain = 0%, 10%, 30%, and 50%); (**e**) I_on_ and g_m, p_ evolution under variable tensional strains for the breathable OECTs; (**f**–**h**) SEM images under 30%, 50%, and 60% strain, respectively.

The gas permeability of devices plays a crucial role in facilitating the exchange of gases or nutrients with the environment, particularly in bioelectronic applications. To evaluate the gas permeability, different samples including blank groups, the gas permeable substrate, the dense substrate, and breathable OECTs were covered tightly with beakers, respectively. Figure 3a presents the results of water vapor transmission for the experimental and reference samples, with water vapor transmission rates of 11.5704, 0.0434, 9.5434, and 7.8693 mg cm^−2^ h^−1^ for the blank group, dense substrate, gas permeable substrate, and breathable OECTs, respectively. The difference in water vapor transmission rate between the gas permeable substrate and OECTs was only 1.6741 cm^−2^ h^−1^, which is attributed to the excellent permeability of the substrate and the scale-matched voids between the active film and substrate [28]. As shown in Figure 3b, the breathable OECTs exhibit a desirable output performance with obvious linear and saturated current states. Figure 3c shows typical p-channel transfer curves, where I_on_ is 0.37 mA, the off-state current (I_off_) is 0.13 μA, and the peak transconductance (g_m, p_) is 1.51 mS. To meet the requirements of next-generation wearable electronics, bioelectronic devices on the skin are required to maintain a high performance at 30% strain [29]. The mechanical stability of the breathable OECTs was evaluated under different uniaxial tensile strains (0%, 10%, 30%, and 50%) applied along the length (L-direction) and width (W-direction) of the channel. In particular, the breathable OECTs were fixed on a stepper motor during tensile testing, where a pre-programmed strain was exerted. The representative transfer curves of the breathable OECTs at various tensile strains were measured and their corresponding I_on_ and g_m_ were summarized, as shown in Figure 3d,e and Table 1. The breathable OECTs exhibited a robust stretchability, as demonstrated in Figure 3d. The transistor characteristics (which include I_on_ and g_m_) for all devices gradually decrease as the stretching strain increases, eventually losing performance at 60% strain due to the P3HT/SEBS film rupture (Figure 3e). Table 1 summarizes the g_m, p_, I_on_ (I_DS_ at gate voltage (V_G_) = −0.9 V and drain voltage (V_D_) = −0.5 V), and threshold voltage (V_th_) for the different stretch states. At 30% strain along the L-direction, V_th-for_ is ~−0.736 V (forward sweep) and V_th-back_ ~−0.659 V (backward sweep), which are almost identical to V_th-for_ ~−0.712 V and V_th-back_ is ~−0.640 V at 30% strain along the W-direction, respectively. This suggests that V_th_ is not significantly affected by the direction of stretching. Nevertheless, when the device undergoes stretching along the L-direction, I_on_ drops from ~0.393 mA in the pristine state to 0.115 mA at 30% strain, but along the W-direction, I_on_ only drops from ~0.454 mA in the pristine state to 0.267 mA at 30% strain. The smaller effect of W-direction stretching can be explained by both that slight cracks are produced along the L-direction and that there is an increase in the W/L ratio. Conversely, L-direction stretching creates cracks perpendicular to the direction of charge transfer and a decreased W/L ratio [30,31]. As expected, g_m, p_ along the L-direction and W-direction in the stretched state follows a similar trend to I_on_ (g_m, p_ along the W-direction changes from 1.860 mS in the pristine state to 0.825 mS at 30% strain and g_m, p_ along the L-direction changes from 1.710 mS in the pristine state to 0.512 mS at 30% strain).

To further determine the reasons for electrical performance degradation, the conductivity of the Au electrode and the interface between the Au electrode and the active layer under different strains was characterized. As shown in Figure S3a and Table S1, the conductance of rectangular electrodes changes from 10.61 mS (pristine state) to 2.26 mS (30% strain) for the length direction, and from 21.47 mS (pristine state) to 2.23 mS (30% strain) for the width direction. As shown in Figure S3b, the stretchability of the 3D porous network Au electrodes was improved compared to the Au electrodes prepared on the flat substrate. However, a mismatched Young’s modulus between the SEBS substrate and the rigid Au electrodes still exists, which leads to Au/SEBS electrode local fracturing during large deformations (Figure S3c–e). In addition, the interface between the electrode and P3HT/SEBS was characterized by optical microscopy under different stretching scenarios, as shown in Figure S4. No significant delamination was observed at 0%, 30%, and 60% strains. Therefore, despite the stretchability of our designed 3D porous network electrodes being improved, the electrical performance of the OECTs still degraded, which is mainly due to the fracture of the Au electrodes under tensile strain.

It is noteworthy that I_on_ and g_m, p_ of the L-direction and W-direction stretches are almost identical at 50% strain, respectively. This is due to at 50% strain, the presence of large cracks plays a dominant role in performance degradation compared to the variation in device geometry (including channel length, channel width, and dielectric thickness). To further understand the mechanisms behind the rapid performance degradation of OECTs under high strain conditions (strain > 50%), the micromorphology of OECTs at different strains (strain = 30%, 50%, and 60%) is shown in Figure 3f–h. It is clear that no cracks are observed at 30% strain. Nevertheless, as the strain increases, fractures first occur in the thinnest walls of the pores due to stress concentration, eventually leading to a large rupture at 60% strain. These results indicate the prepared OECTs devices not only have excellent gas permeability, but also maintain operation under 30% tensile deformation.

### 3.3. Sensitive Detection of Glucose

**Figure 4:**
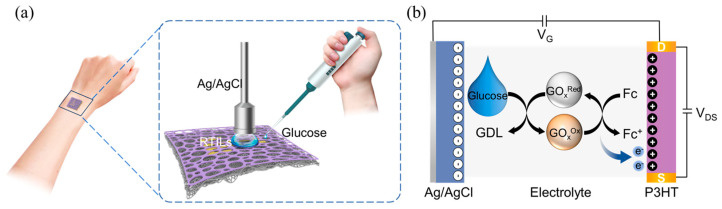
Breathable glucose sensors with the OECTs. (**a**) Schematic of the process as a glucose sensor; (**b**) the working mechanism of the glucose sensor.

RTILs have been used as replacements for aqueous electrolytes such as PBS due to their large electrochemical operating window and high ionic conductivity [32]. In particular, while RTILs have been used as a solvent for enzymes, the enzyme is able to maintain its selectivity and high stability, even enhancing the catalytic activity [33,34]. Thus, EMIM:TFSI was used as a reservoir for GOx. The operating procedure for glucose detection is shown in Figure 4a and details of the procedure are shown in the characterization section. The channel region was filled with EMIM:TFSI containing GOx and Fc, and a glucose solution was introduced into the channel region after I_DS_ stabilized. Previous studies confirmed that the dissolution of enzymes in an RTIL can lead to changes in the secondary and primary structure of the enzyme and result in the loss of enzyme activity [35,36]. In contrast to Fc, which readily dissolves in EMIM:TFSI, GOx exists in a dispersed state and dissolves only when reacting with glucose solution. The operating mechanism of the glucose sensor is shown in Figure 4b. In the electrolyte, GOx oxidizes glucose to gluconolactone (GDL) and produces the intermediate product, H_2_O_2_ molecules (Equation (1)). The generated H_2_O_2_ molecules are further oxidized to protons and electrons under the catalysis of Fc (Equation (2)) [37].

(1) $$D\text{-}\mathrm{Glucose}\to D\text{-}\mathrm{Gluconolactone}+O_{2}$$

(2) $$H_{2}O_{2}+O_{2}\to 2H^{+}+2e^{-}$$

The electrons generated from oxidation process are transferred to the channel under a negative V_G_, which reduces the carrier density of P3HT through electron–hole recombination, resulting in a decrease in I_DS_. Therefore, the I_DS_ reduction strongly correlates with an increase in glucose concentration, which could be applied in glucose detection. 

**Figure 5:**
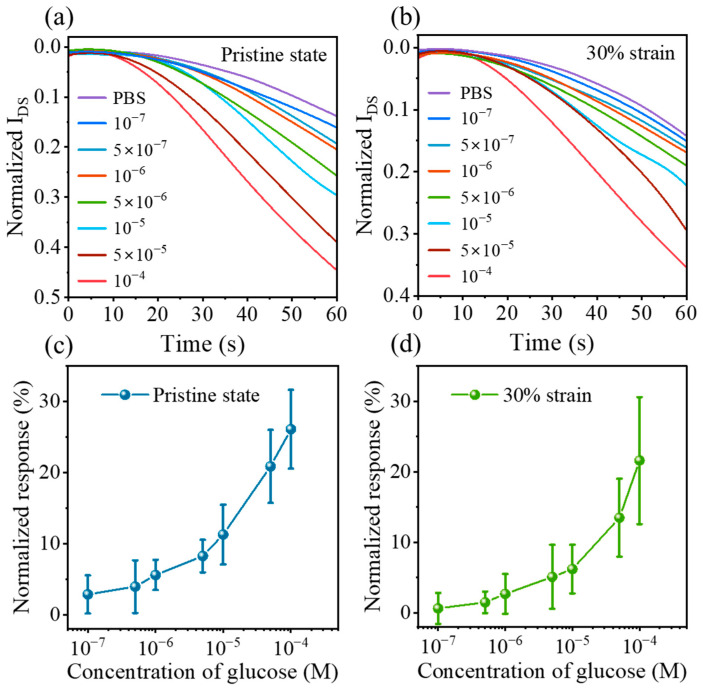
Glucose sensor performance under different operating conditions. Normalized currents after injection of different concentrations of glucose in the pristine state (**a**) and 30% strain (**b**); normalized response curves of OECTs to glucose concentration at 0% (**c**) and 30% strain (**d**) (V_G_ = −0.7 V, V_D_ = −0.5 V).

In order to compare the variation in *I_DS_* after glucose injection, the normalized current (*NC*) was obtained by the following Equation (3):

(3) $$NC=\left|\left.\frac{I_{DS}^{Conc=c}}{I_{DS}^{CONC=0}}\right|\right.$$

where $I_{DS}^{Conc=c}$ and $I_{DS}^{CONC=0}$ are the drain currents before and after glucose injection, respectively. Figure 5a,b shows the variation in *NC* with glucose concentration for OECT devices at 0% and 30% strain, respectively, where the 30% strain is applied along the W-direction. Specifically, a wide range of concentrations from 10^−7^ to 10^−4^ M can be detected under both 0% and 30% tensile strain, covering the clinical glucose level in human saliva (8 × 10^−6^–2.1 × 10^−4^ M) and sweat (5 × 10^−5^–2 × 10^−4^ M). Figure 5c,d shows the normalized response (Δ*I_DS_*/*I*_*DS*0_) variation in glucose concentration for pristine and 30% stretched devices. The normalized response of the glucose sensor under 30% strain declines compared to the pristine device (from ~26% to ~21% at 10^−4^ M), which is attributed to the reduction in electrical properties under tension. These results show that the device can respond to glucose significantly and realize the mechanical robustness required for wearable electronics (~30% deformation), sufficiently detecting glucose concentrations present in human body fluids, like saliva and sweat.

**Figure 6:**
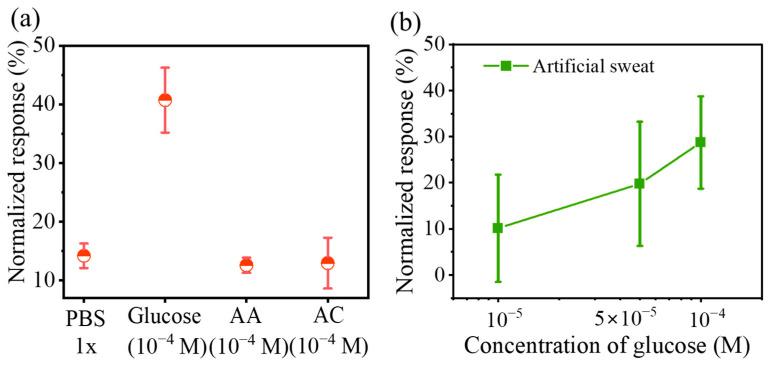
(**a**) Selectivity of the OECTs sensor to glucose, uric acid (UA), ascorbic acid (AA), glucose, and pure PBS; (**b**) normalized response of the glucose sensor to artificial sweat.

A crucial aspect lies in the comparison of biosensor selective performance, which serves as the key factor in biosensor accuracy. Thus, we have performed an assessment of the glucose sensor’s selective characteristics against uric acid (UA), ascorbic acid (AA), and pure PBS solution, as shown in Figure 6a. Notably, when compared to its performance with glucose (10^−4^ M), the breathable sensors display negligible responses in the presence of UA (10^−4^ M), AA (10^−4^ M), and the blank control group (1 × PBS). The slight interference current can be addressed by using a blank channel or gate via common circuit engineering approaches. We employed artificial sweat containing different concentrations of glucose (1 × 10^−5^, 5 × 10^−5^, and 1 × 10^−4^ M) to demonstrate the potential application of our devices. As shown in Figure 6b, the breathable sensors successfully detect artificial sweat containing varying concentrations of glucose. Meanwhile, these outcomes align with the results obtained from our previous experiments. These results illustrate the potential application of the highly breathable and stretchable glucose sensors in wearable electronics systems, such as skin adhesive patches, epidermal electronics, and smart e-skin.

## 4. Conclusions

In summary, we successfully prepared breathable OECT sensors for glucose sensing. By laminating a breathable SEBS substrate with a porous P3HT/SEBS active film, this unique combination enabled OECT to exhibit a remarkable breathability. The porous elastic structure enables them to achieve up to 30% stretching while maintaining excellent electrical stability. In this research, we also achieved the sensitive detection of glucose using these devices. The glucose detection capability of the OECTs spanned a wide concentration range from 10^−7^ M to 10^−4^ M, even under 30% strain. In addition, the reported breathable sensors not only provide a simple approach to improve the wearing comfort and sensing ability, but also show promise for non-invasive and real-time monitoring applications.

## Figures and Tables

**Table 1:** OECTs performance indicators under different tensile strain values (V_D_ = −0.5 V).

Direction of Strain	Range of Strain (%)	g_m, p_ (mS)	I_on_ (mA)	V_th-for_ (V)	V_th-back_ (V)
L-direction	0	1.710 ± 0.458	0.393 ± 0.099	−0.711 ± 0.008	−0.642 ± 0.010
	10	0.984 ± 0.075	0.239 ± 0.011	−0.703 ± 0.002	−0.635 ± 0.017
	20	0.539 ± 0.164	0.123 ± 0.036	−0.726 ± 0.017	−0.639 ± 0.015
	30	0.512 ± 0.177	0.115 ± 0.042	−0.736 ± 0.005	−0.659 ± 0.015
	40	0.394 ± 0.295	0.090 ± 0.072	−0.725 ± 0.013	−0.644 ± 0.004
	50	0.352 ± 0.281	0.081 ± 0.065	−0.729 ± 0.006	−0.660 ± 0.058
W-direction	0	1.860 ± 0.289	0.454 ± 0.069	−0.711 ± 0.007	−0.636 ± 0.004
	10	1.670 ± 0.311	0.407 ± 0.073	−0.718 ± 0.004	−0.635 ± 0.008
	20	1.500 ± 0.315	0.368 ± 0.076	−0.710 ± 0.005	−0.640 ± 0.011
	30	0.825 ± 0.631	0.267 ± 0.103	−0.712 ± 0.006	−0.640 ± 0.010
	40	0.632 ± 0.330	0.151 ± 0.087	−0.712 ± 0.023	−0.647 ± 0.028
	50	0.362 ± 0.287	0.083 ± 0.071	−0.731 ± 0.008	−0.650 ± 0.022

## Data Availability

Not applicable.

## Supplementary Materials

The following supporting information can be downloaded at: https://www.mdpi.com/article/10.3390/s23156910/s1, Figure S1: The molecular structures of P3HT, SEBS, and EMIM: TFSI; Figure S2: The cross-sectional SEM image of the SEBS substrate; Figure S3: Mechanical characteristics of Au electrodes; Figure S4: The optical images of the interface between P3HT/SEBS film and the Au electrode under 0%, 30%, and 60% strain; Table S1: The conductance of porous network Au electrodes under different strains.
