# Supplementary material for: Breathable and Stretchable Organic Electrochemical Transistors with Laminated Porous Structures for Glucose Sensing
Source: Sensors (Basel). 2023 Aug 3;23(15):6910. doi: 10.3390/s23156910 (PMC10422285; doi:10.3390/s23156910)
Supplement: Supplementary file 1 [file sensors-23-06910-s001.zip › sensors-2521340-supplementary.pdf]

Supporting Information

Breathable and stretchable organic electrochemical transistors with laminated porous structures for glucose sensing

Haihong Guo, Changjian Liu, Yujie Peng, Lin Gao, Junsheng Yu *

State Key Laboratory of Electronic Thin Films and Integrated Devices, School of Optoelectronic Science and Engineering, University of Electronic Science and Technology of China (UESTC), Chengdu 610054, China; 202221050501@std.uestc.edu.cn (H.G.); 202121050109@std.uestc.edu.cn (C.L.); 202211050804@std.uestc.edu.cn (Y.P.); 202111050704@std.uestc.edu.cn (L.G.)

* Correspondence: jsyu@uestc.edu.cn

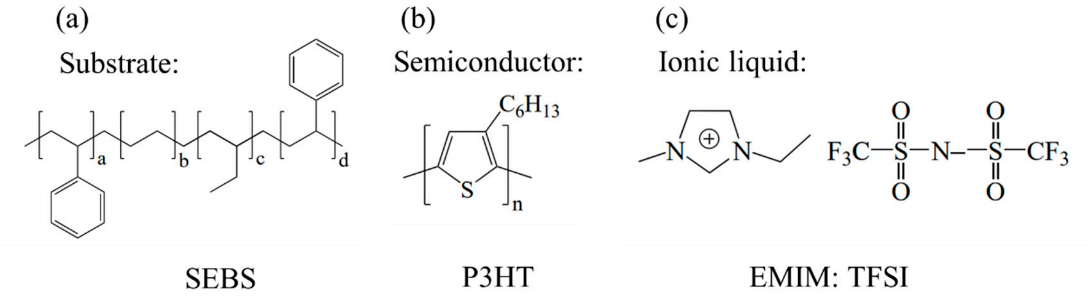

Figure S1. The molecular structures of P3HT, SEBS and EMIM: TFSI.

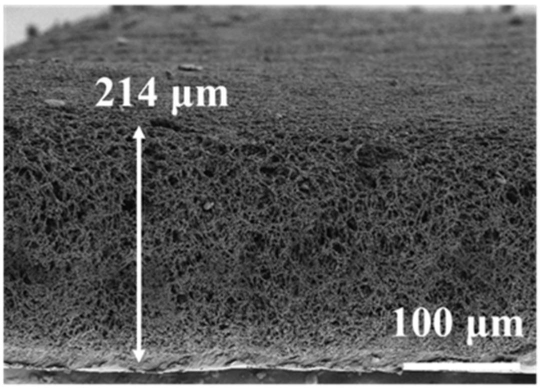

Figure S2. The Cross-sectional SEM image of SEBS substrate.

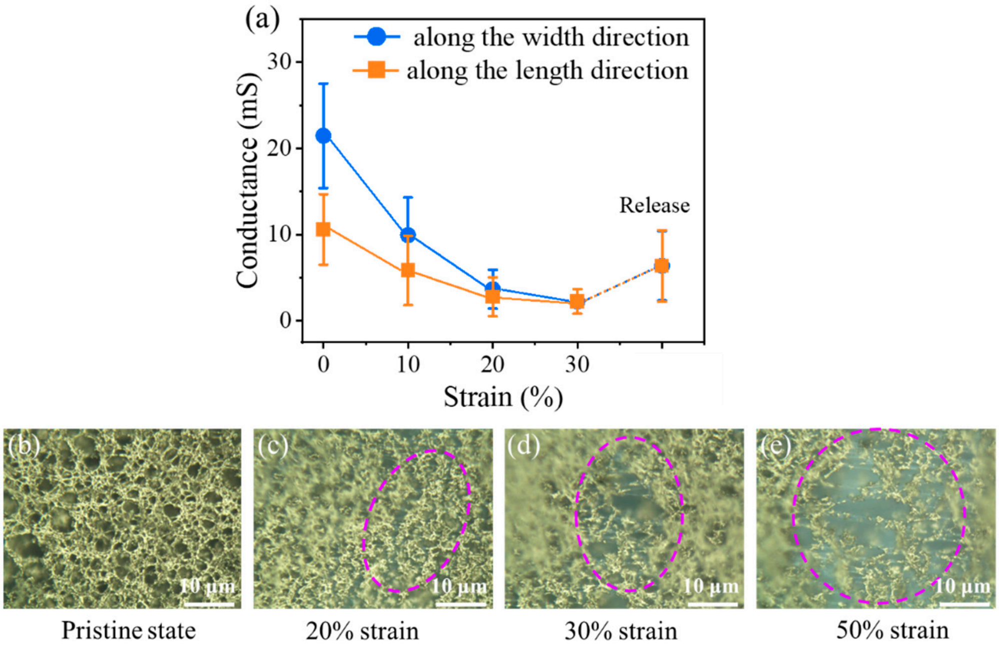

Figure S3. Mechanical characteristics of Au electrodes. (a) Conductance of porous network Au electrodes under different strains (10%, 20%, 30% strain and release) along the channel length and width directions; (b-e) the optical images of porous network Au electrodes under different strains (0%, 20%, 30% and 50% strain).

Table S1. Conductance of porous network Au electrodes under different strains.

Direction of strain	Tensile strength (%)	Conductance (mS)
Length direction	0	10.61±4.1
	10	5.85±4.02
	20	2.79±2.26
	30	2.26±1.43
	Release	6.38±4.15
Width direction	0	21.47±6.05
	10	9.95±4.37
	20	3.67±2.26
	30	2.23±0.41
	release	6.39±4.03

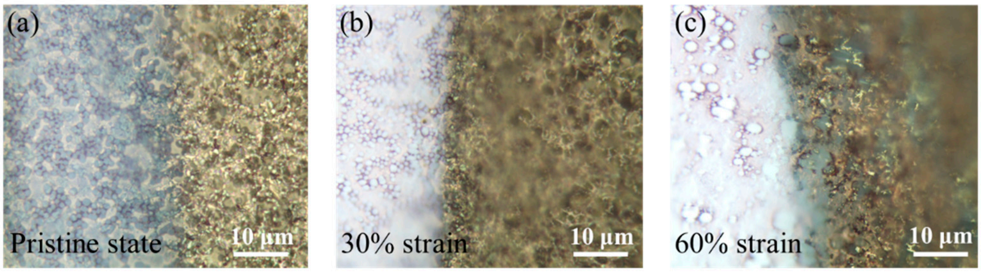

Figure S4. The optical images of the interface between P3HT/SEBS film and Au electrode under 0%, 30% and 60% strain.
